# Supplementary material for: Thermocatalytic hydrogen peroxide generation and environmental disinfection by Bi2Te3 nanoplates
Source: Nat Commun. 2021 Jan 8;12:180. doi: 10.1038/s41467-020-20445-0 (PMC7794375; doi:10.1038/s41467-020-20445-0)
Supplement: Supplementary file 1 — Supplementary Information [file 41467_2020_20445_MOESM1_ESM.pdf]

Supplementary Information

Thermocatalytic Hydrogen Peroxide Generation and Environmental Disinfection by Bi_2Te_3 Nanoplates

Yu-Jiung Lin[1], Imran Khan[2], Subhajit Saha[1], Chih-Cheng Wu[1,3,4,5], Snigdha Roy Barman[1], Fu-Cheng Kao[1,6] and Zong-Hong Lin[1,7,8*]

[1] Institute of Biomedical Engineering, National Tsing Hua University, Hsinchu 30013, Taiwan

[2] Institute of NanoEngineering and Microsystems, National Tsing Hua University, Hsinchu 30013, Taiwan

[3] Cardiovascular Center, National Taiwan University Hospital, Hsinchu Branch, Hsinchu 30059, Taiwan

[4] College of Medicine, National Taiwan University, Taipei 10051, Taiwan

[5] Institute of Cellular and System Medicine, National Health Research Institute, Zhunan 35053, Taiwan

[6] Department of Orthopaedic Surgery, Spine Section, Chang Gung Memorial Hospital, Taoyuan 33305, Taiwan

[7] Department of Power Mechanical Engineering, National Tsing Hua University, Hsinchu 30013, Taiwan

[8] Frontier Research Center on Fundamental and Applied Sciences of Matters, National Tsing Hua University, Hsinchu 30013, Taiwan

These authors contributed equally: Yu-Jiung Lin, Imran Khan

[*] Corresponding author: linzh@mx.nthu.edu.tw

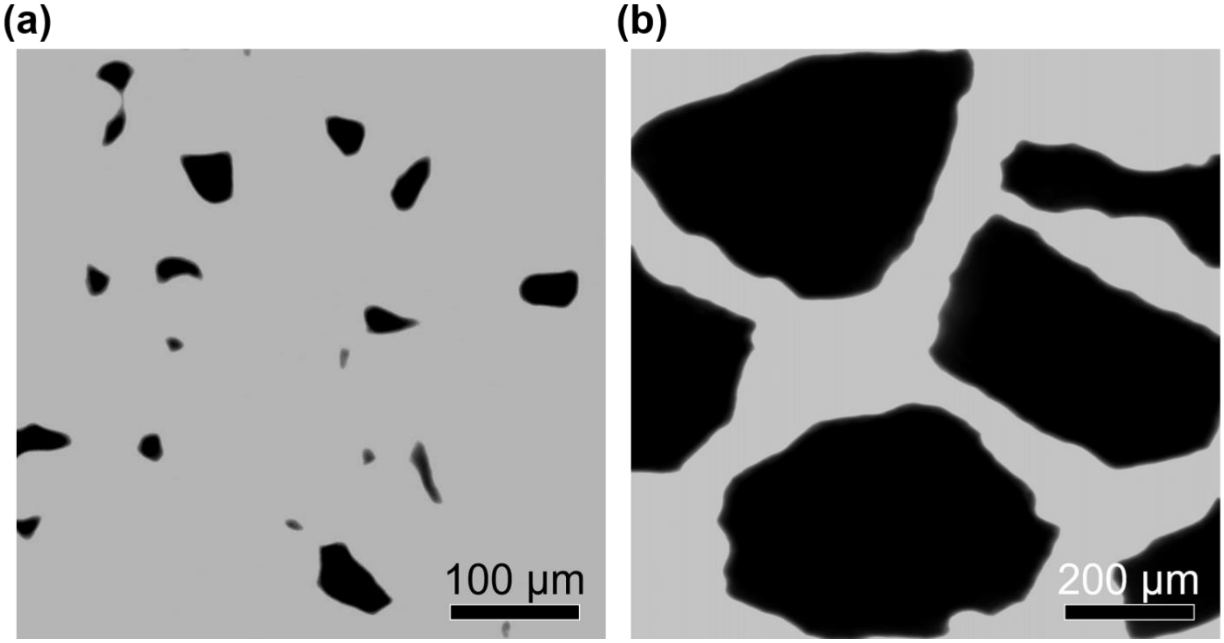

Supplementary Fig. 1 Optical microscope images of bulk **a** Bi_2Te_3 and **b** Sb_2Te_3 thermoelectric materials.

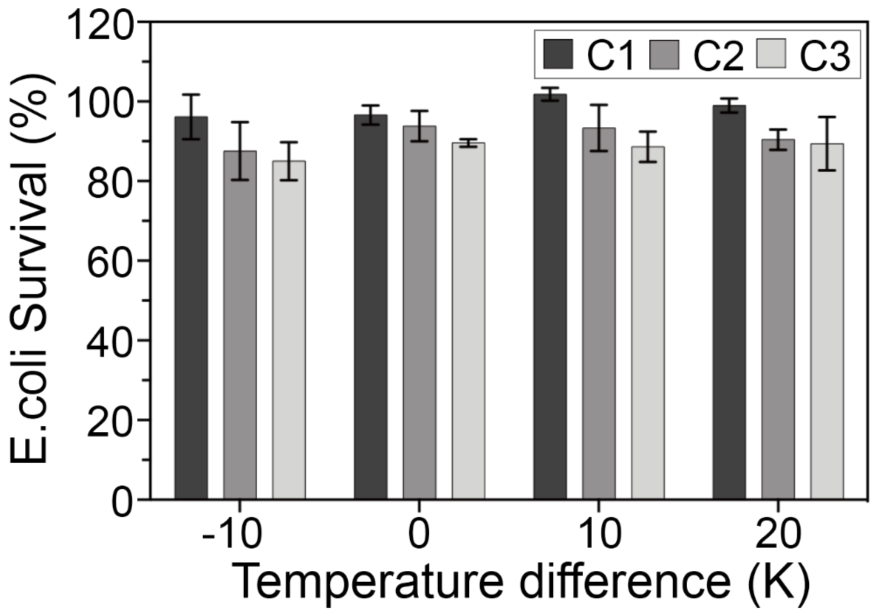

Supplementary Fig. 2 Control experiment of pure *E. coli* (without any catalyst) under different temperature differences and three thermal cycles such as C1, C2 and C3.

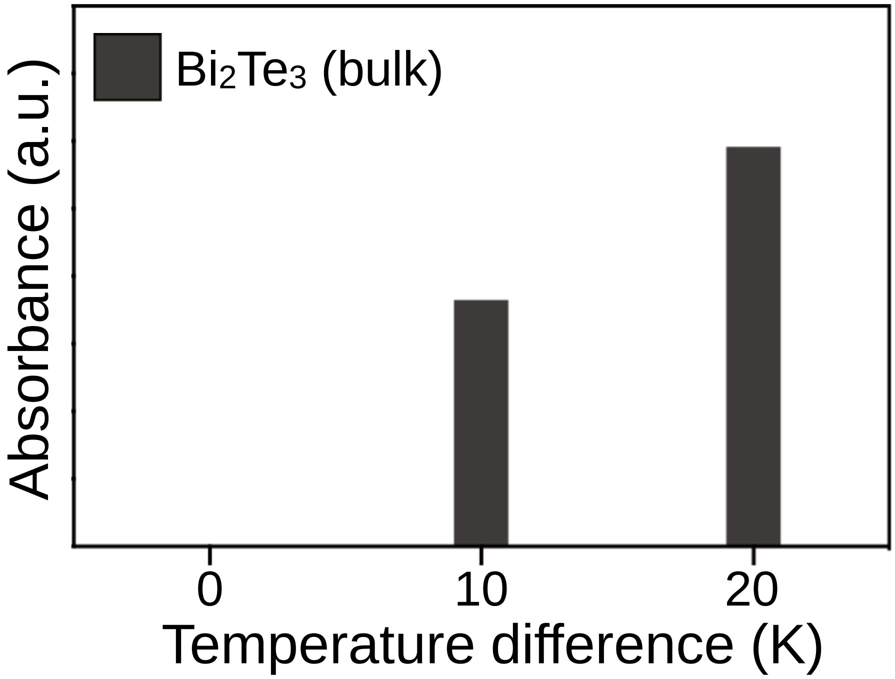

Supplementary Fig. 3 $\cdot O_2$ generation by bulk Bi_2Te_3 (50 mg) under different temperature differences.

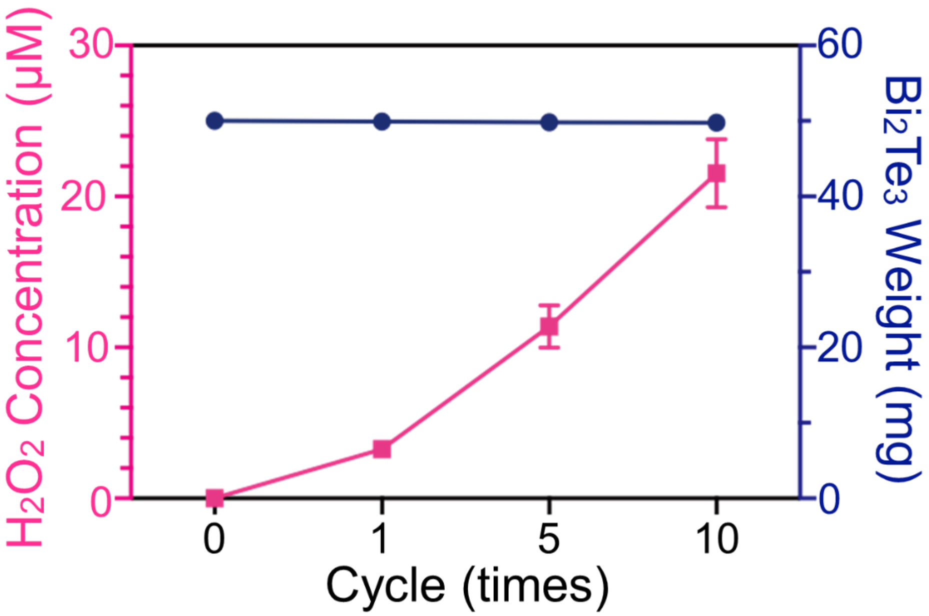

Supplementary Fig. 4 Quantification of H_2O_2 generation by bulk Bi_2Te_3 (50 mg) and the weight of bulk Bi_2Te_3 at different thermal cycles.

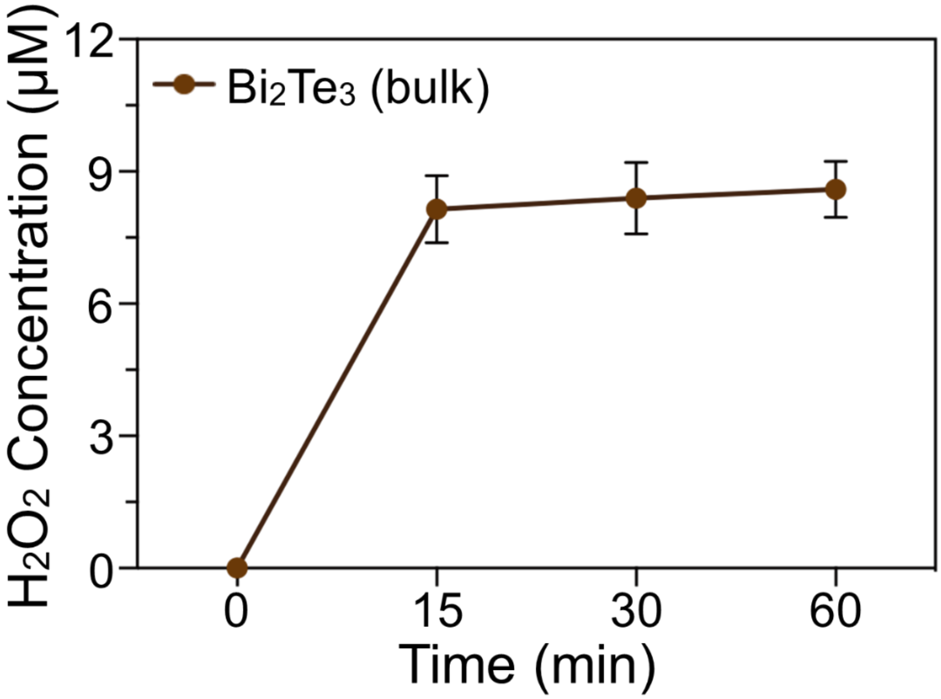

Supplementary Fig. 5 H_2O_2 generation by bulk Bi_2Te_3 (50 mg). First, the temperature difference was applied for 15 min, and after that, the surrounding temperature was kept constant to observe the effect of temperature difference on H_2O_2 generation.

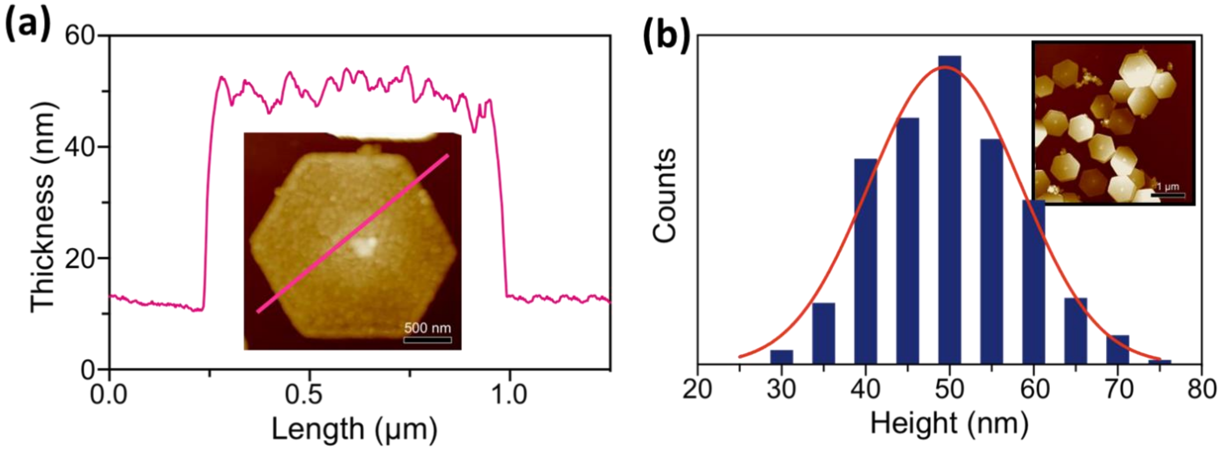

Supplementary Fig. 6 a The thickness profile of a single Bi_2Te_3 nanoplate analyzed by AFM; **b** The thickness distribution of Bi_2Te_3 nanoplates calculated from the AFM data.

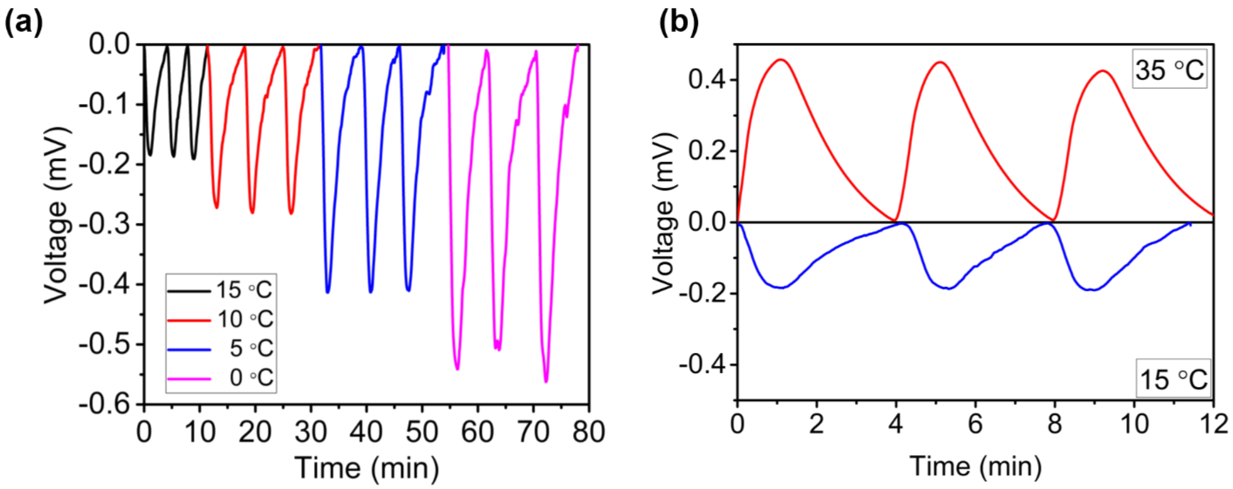

Supplementary Fig. 7 **a** Representation of thermoelectric voltage generation by Bi_2Te_3 NPs at different temperatures below room temperature. **b** Comparison of thermoelectric voltage generated by Bi_2Te_3 NPs at 35 °C and 15 °C corresponding to +10 K and -10 K temperature difference, respectively.

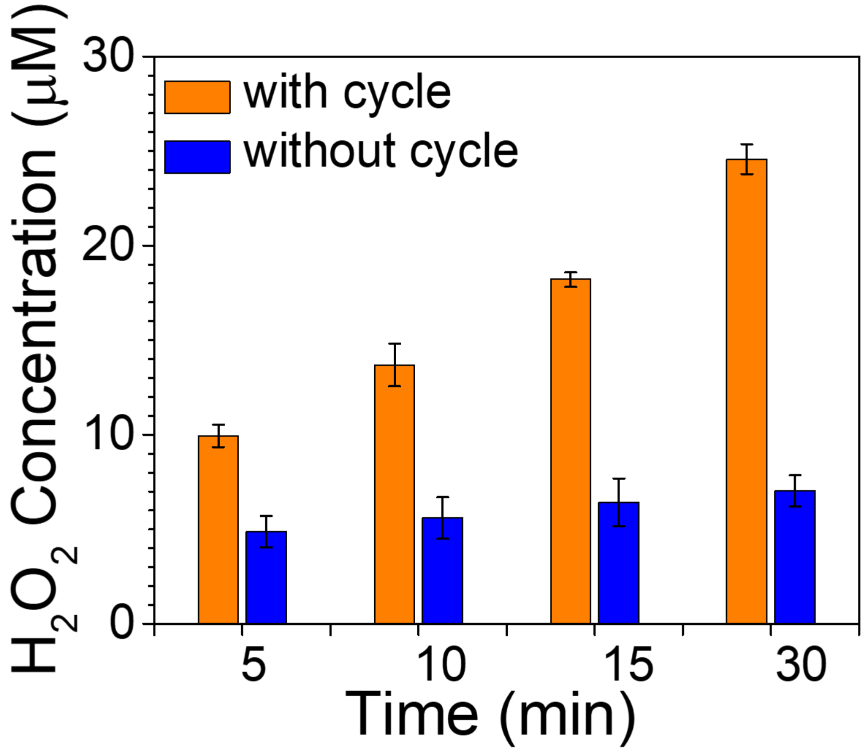

Supplementary Fig. 8 Comparison of thermocatalytic H_2O_2 generation by Bi_2Te_3 NPs at +10 K temperature difference in cyclic heating and continuous heating conditions.

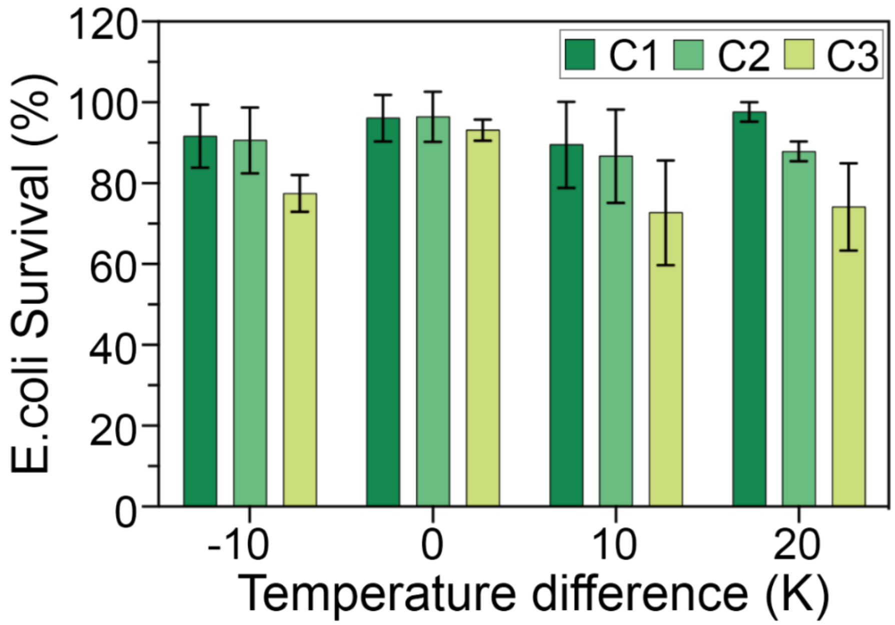

Supplementary Fig. 9 Disinfection performance of bulk Bi_2Te_3 (5 mg) under different temperature differences and cycles.

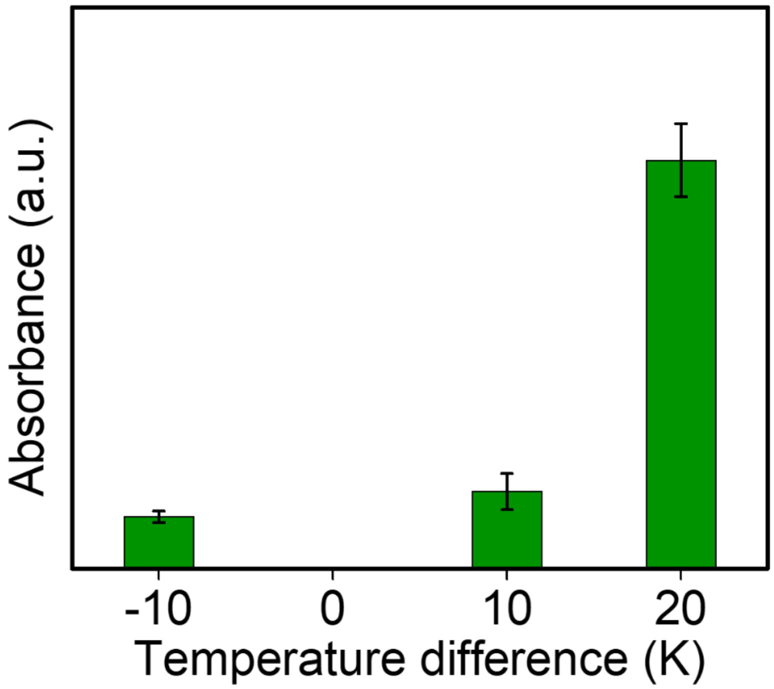

Supplementary Fig. 10 Demonstration of $\cdot O_2$ generation by Bi_2Te_3 NPs under different temperature differences.

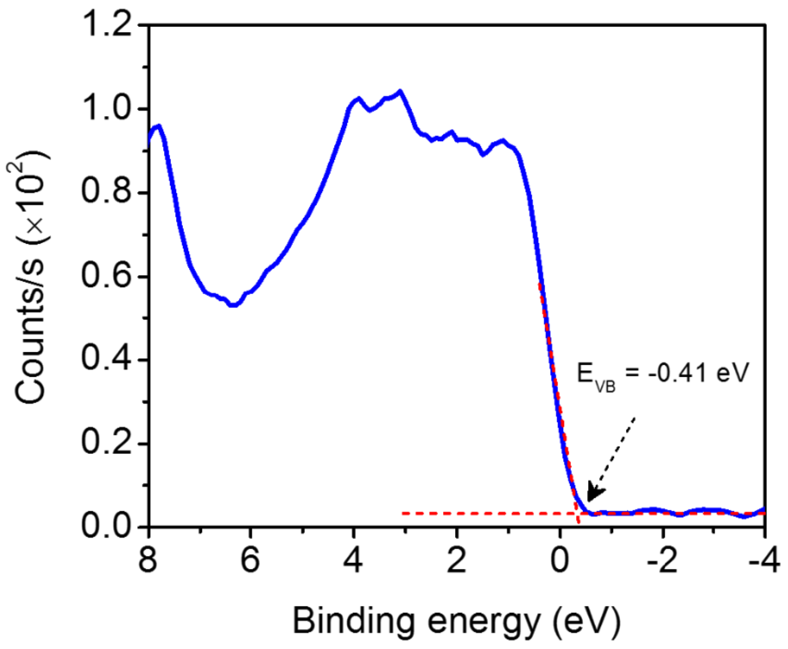

Supplementary Fig. 11 Valence band XPS spectra of Bi_2Te_3 NPs representing the valence band maximum (VBM) position.

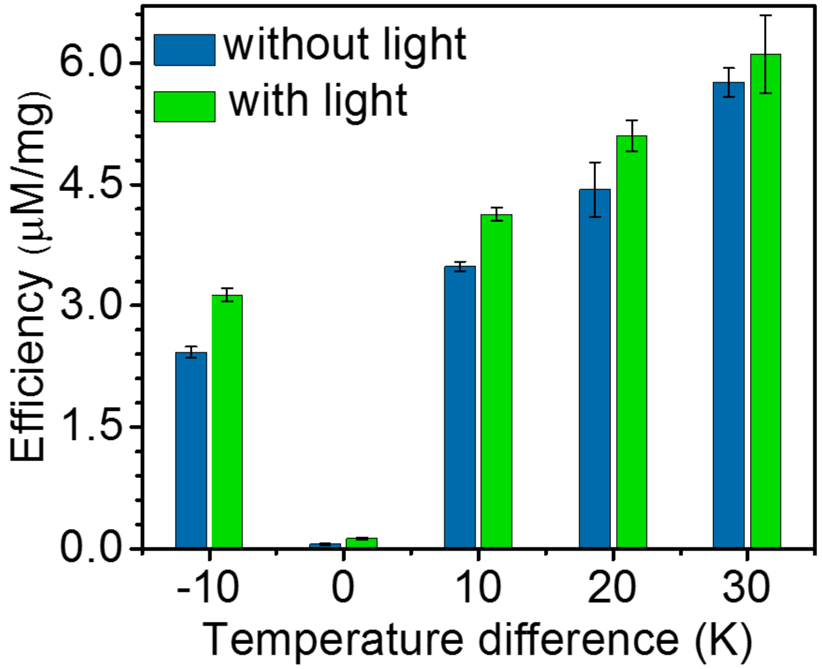

Supplementary Fig. 12 Comparison of H_2O_2 generation performance by Bi_2Te_3 NPs under dark and ambient light conditions.

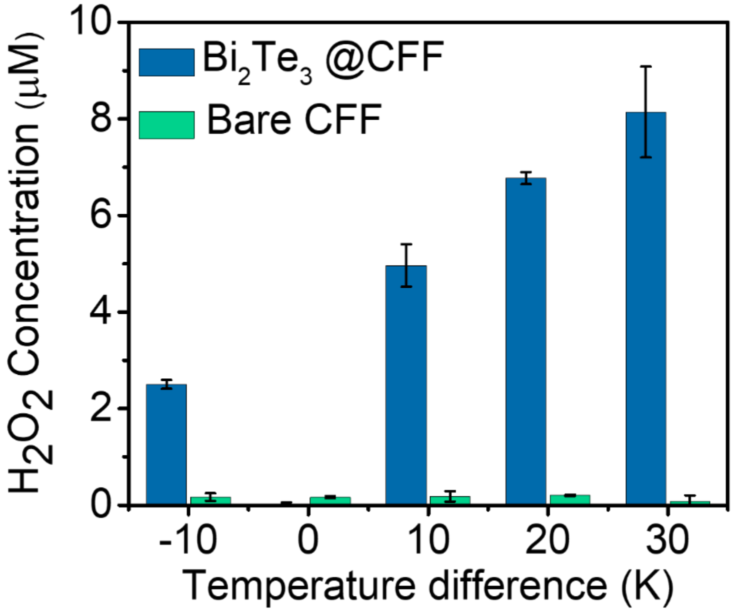

Supplementary Fig. 13 Comparison of H_2O_2 generation efficiency demonstrated by Bi_2Te_3@CFF and bare CFF.

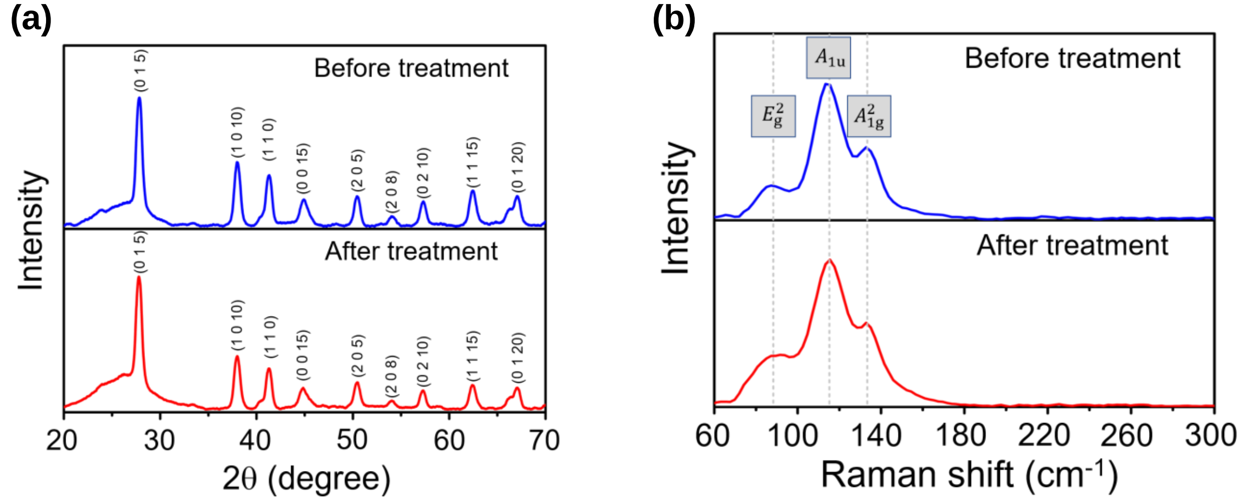

Supplementary Fig. 14 XRD patterns **a** and Raman spectra **b** of Bi_2Te_3@CFF before and after 30 days of thermocatalytic reaction on air conditioner.
