# Supplementary material for: Thermocatalytic hydrogen peroxide generation and environmental disinfection by Bi2Te3 nanoplates
Source: Nat Commun. 2021 Jan 8;12:180. doi: 10.1038/s41467-020-20445-0 (PMC7794375; doi:10.1038/s41467-020-20445-0)
Supplement: Supplementary file 3 — Reporting Summary [file 41467_2020_20445_MOESM3_ESM.pdf]

nature research

Corresponding author(s): DBPR NCOMMS-20-24018B

Last updated by author(s): Dec 1, 2020

Reporting Summary

Nature Research wishes to improve the reproducibility of the work that we publish. This form provides structure for consistency and transparency in reporting. For further information on Nature Research policies, see our Editorial Policies and the Editorial Policy Checklist.

Statistics

For all statistical analyses, confirm that the following items are present in the figure legend, table legend, main text, or Methods section.

n/a	Confirmed	
☐	☒	The exact sample size (*n*) for each experimental group/condition, given as a discrete number and unit of measurement
☐	☒	A statement on whether measurements were taken from distinct samples or whether the same sample was measured repeatedly
☒	☐	The statistical test(s) used AND whether they are one- or two-sided<br>*Only common tests should be described solely by name; describe more complex techniques in the Methods section.*
☒	☐	A description of all covariates tested
☒	☐	A description of any assumptions or corrections, such as tests of normality and adjustment for multiple comparisons
☒	☐	A full description of the statistical parameters including central tendency (e.g. means) or other basic estimates (e.g. regression coefficient) AND variation (e.g. standard deviation) or associated estimates of uncertainty (e.g. confidence intervals)
☒	☐	For null hypothesis testing, the test statistic (e.g. F, t, r) with confidence intervals, effect sizes, degrees of freedom and P value noted<br>*Give P values as exact values whenever suitable.*
☒	☐	For Bayesian analysis, information on the choice of priors and Markov chain Monte Carlo settings
☒	☐	For hierarchical and complex designs, identification of the appropriate level for tests and full reporting of outcomes
☒	☐	Estimates of effect sizes (e.g. Cohen's *d*, Pearson's *r*), indicating how they were calculated

Our web collection on statistics for biologists contains articles on many of the points above.

Software and code

Policy information about availability of computer code

Data collection	No software was used
Data analysis	Origin Pro, Microsoft Office

For manuscripts utilizing custom algorithms or software that are central to the research but not yet described in published literature, software must be made available to editors and reviewers. We strongly encourage code deposition in a community repository (e.g. GitHub). See the Nature Research guidelines for submitting code & software for further information.

Data

Policy information about availability of data

All manuscripts must include a data availability statement. This statement should provide the following information, where applicable:

- Accession codes, unique identifiers, or web links for publicly available datasets
- A list of figures that have associated raw data
- A description of any restrictions on data availability

Source Data are provided with this paper. All the experimental results of the main manuscript and Supplementary Information are available in Figshare repository via the identifier https://figshare.com/s/e2460939d5f21d4aefdc.

Field-specific reporting

Ecological, evolutionary & environmental sciences study design

All studies must disclose on these points even when the disclosure is negative.

Study description	Temperature difference induced generation of reactive oxygen species by thermoelectric materials and its antibacterial application.
Research sample	Thermocatalytic ROS generation by two group of samples e.g. bulk and nanoparticles are reported. The nanometric Bi2Te3 is further coated on carbon fiber fabric to design the antibacterial filter.
Sampling strategy	Three batch of the samples were used to conduct the experiments and the consequent error bars are provided in the experimental results.
Data collection	Phase purity and surface morphology were investigated by XRD and FESEM/TEM. Elemental analysis were carried out by EDX attached with TEM. Temperature dependent surface potential distributions were measured by KPFM technique. Band position of the materials were determined by VB-XPS technique. Thermoelectric voltages were recorded with a low noise voltage preamplifier. Thermocatalysis experiments were conducted in a water bath. Amplex Red with HRP enzyme was used for H2O2 detection. The thermocatalytic disinfection tests were conducted using a hair dryer and a cooling fan. Real-time application of the antibacterial filter was demonstrated via domestic air-conditioner. Y-J.L., I.K. designed the experiments and carried out the material synthesis and device fabrication. S.R.B. and C-C.W. conducted the antibacterial experiments. Biocompatibility tests were carried out by F-C.K.
Timing and spatial scale	All the data were recorded within January, 2018 and September, 2020.
Data exclusions	No data were excluded from the analyses.
Reproducibility	Reproducibility of experimental findings were checked and it was found that all the data were well-reproducible.
Randomization	Since only two group of samples e.g. bulk and nanometric thermoelectric materials were used in the experiments; randomization is not relevant in our case.
Blinding	Control experiments were performed to establish/ support the findings.

Did the study involve field work? ☐ Yes ☒ No

Reporting for specific materials, systems and methods

We require information from authors about some types of materials, experimental systems and methods used in many studies. Here, indicate whether each material, system or method listed is relevant to your study. If you are not sure if a list item applies to your research, read the appropriate section before selecting a response.

Materials & experimental systems

n/a	Involved in the study	
☒	☐	Antibodies
☒	☐	Eukaryotic cell lines
☒	☐	Palaeontology and archaeology
☐	☒	Animals and other organisms
☒	☐	Human research participants
☒	☐	Clinical data
☒	☐	Dual use research of concern

Methods

n/a	Involved in the study	
☒	☐	ChIP-seq
☒	☐	Flow cytometry
☒	☐	MRI-based neuroimaging

Animals and other organisms

Policy information about studies involving animals; ARRIVE guidelines recommended for reporting animal research

Laboratory animals	Species-Mice, Strain-BalB/c, Sex-Female, Age-6 weeks
Wild animals	This study did not involve wild animals.
Field-collected samples	This study did not involve samples collected from the field.
Ethics oversight	Animal use protocol has been reviewed and approved by the Institutional Animal Care and Use Committee (IACUC), and the committee recognises that the proposed animal experiment follows the Animal Protection Law by the Council of Agriculture Executive Yuan, R.O.C and the guidelines as shown in the Guide for the Care and Use of Laboratory Animals as promulgated by the Institute of Laboratory Animal Resources, National Research Council, U.S.A.

Note that full information on the approval of the study protocol must also be provided in the manuscript.
